# Thirty Years of Compositional Change in an Old-Growth Temperate Forest: The Role of Topographic Gradients in Oak-Maple Dynamics

**DOI:** 10.1371/journal.pone.0160238

**Published:** 2016-07-28

**Authors:** Julia I. Chapman, Ryan W. McEwan

**Affiliations:** Department of Biology, University of Dayton, Dayton, Ohio, United States of America; Chinese Academy of Sciences, CHINA

## Abstract

Ecological communities are structured in response to spatial and temporal variation of numerous factors, including edaphic conditions, biotic interactions, climatic patterns and disturbance regimes. Widespread anthropogenic factors such as timber harvesting can create long-lasting impacts, obscuring the relationship between community structure and environmental conditions. Minimally impacted systems such as old-growth forests can serve as a useful ecological baseline for predicting long-term compositional shifts. We utilized decadal tree species sampling data (1979–2010) divided into three strata (understory, midstory, overstory) to examine temporal changes in relative abundances and spatial distributions of dominant taxa, as well as overall shifts in community composition, in a relatively pristine Appalachian old-growth forest in eastern Kentucky, USA. *Quercus* and *Carya* species persisted mainly as mature canopy trees with decreasing juvenile recruitment, especially in mesic areas. In contrast, *Acer*, *Fagus*, and other mesophytic species were abundant and spatially widespread in subcanopy layers suggesting these species are more likely to recruit in gap-scale canopy openings. In the overstory, mesophytic species were spatially restricted to lower and mid-slope mesic habitats. Temporal changes in community composition were most evident in the understory and tended to be greater in mesic areas, a trend seemingly driven by recruitment failure among xerophytic species. In subcanopy vegetation we discovered a loss of distinction through time among the ecological community designations established following the 1979 survey (Chestnut oak, Mixed mesophytic, and Beech). The overstory was more stable through time, suggesting a storage effect where long-lived trees have maintained a particular community composition through time in areas where regeneration opportunities are minimal under current environmental conditions. Overall, sitewide canopy succession is occurring slowly in the absence of major disturbance, and topography-driven environmental variation appears to have an important local-scale filtering effect on communities.

## Introduction

In the face of widespread and increasing anthropogenic activity, the ability to understand and predict ecosystem changes has become a central goal in ecology. Forest ecosystems are of particular interest due to their critical role in global biogeochemical cycles, provision of ecosystem services, and potential for mitigating climate change [1,2]. Although forest compositional data are abundant, it can be difficult to place findings within the theoretical framework of forest community dynamics because there are numerous (and possibly competing) mechanisms proposed to explain compositional changes through time. Observational studies documenting baseline community composition, structure and dynamics, especially in areas of minimal anthropogenic impact, are valuable reality-based starting points for ecological models as well as historical records for future assessments of ecosystem change. Meaningful application of such datasets requires an understanding of the inherent patterns in the data as well as their driving mechanisms—ecological communities are complex systems that require equally sophisticated explanations.

In mature forests, turnover among overstory (canopy) trees is largely driven by gap formation resulting from weather disturbance or mortality events [3–5] and occurs at a rate of around 1% per year in eastern North American deciduous forests [6–8]. This slow rate of canopy replacement coupled with the long lifespans of tree species in this region results in a ‘storage effect’ [9] where a particular overstory composition can be maintained for several hundred years even through changes in environmental conditions or disturbance regimes. Recruitment and mortality of subcanopy trees, however, takes place on decadal or even annual timeframes and reflects a combination of typical forest stand dynamics [10] and environmental stochasticity. Recruitment opportunities for seedlings, saplings and small trees in canopy gaps may be approximated by the “lottery model”, which in its most simple form operates on a “first-come, first-serve” basis where all individuals have an equal chance of capturing resource space [11,12]. In reality, the outcomes of such recruitment events are dependent upon the composition and demography of juvenile populations, which can be highly variable through space and time [13–17] due to factors such as habitat heterogeneity, dispersal limitation, environmental fluctuations, growth response characteristics, and density-dependent interactions such as competition [18–21]. This variability combined with the stochastic nature of canopy gap formation is thought to allow sufficient recruitment opportunities for a wide range of species [16]; however, species with consistently poor juvenile establishment and survival (recruitment-limited) can be at a disadvantage [19,22]. The relative abundances of species can affect “lottery” outcomes as more abundant species are more likely to capture available resources [23], and over the long term, this could result in broad-scale compositional shifts.

Mature deciduous forests in eastern North America have been the subject of numerous studies on compositional change, focusing largely on the recent broad-scale shift in species composition, termed “mesophication” [24], where oaks (*Quercus* spp.) are failing to regenerate or recruit and are being replaced or outcompeted by an alternate suite of species, primarily maples (*Acer rubrum* L. and *A*. *saccharum* Marshall; e.g.,[25–45]). Much effort has gone into determining the drivers of this shift including, but not limited to, altered fire regime, regional climate shifts, and changing herbivore densities [24,46–48], but there is also opportunity to examine fundamental mechanisms of forest succession (storage effect, lottery, recruitment limitation) in these systems. Long-term changes have already documented been documented in several mature and old-growth forests (e.g. [34,40,41,49,50]), but there is a need to better describe the local-scale spatial patterns of forest turnover within ecologically meaningful strata (*i*.*e*., stem size classes). Such analyses may reveal more about the role of stochasticity and environmental filtering in recruitment and better elucidate the successional trajectory of the Eastern Deciduous Forests.

A topographically complex Appalachian old-growth forest located within the Lilley Cornett Woods Appalachian Research Station provided an opportunity to assess patterns and drivers of long-term temperate forest dynamics. This site has a rich history of ecological research documenting forest community dynamics through space and time and in relation to edaphic variation at the site [51–54]. One particularly interesting notion derived from this work, which has been similarly concluded in other studies (e.g. [32,55–57]), is that oak-maple recruitment dynamics at this site are spatially patterned relative to topography such that oak recruitment is strongest in the most xeric areas of the watershed and maples are recruited most strongly at mid-elevations [52]. The overarching objective of this project was to enhance our understanding of these recruitment dynamics by using 30 years of decadal tree sampling data (1979–2010) divided into three relevant forest strata (understory, midstory, overstory), and to link mechanisms of forest community assembly to spatial and temporal patterns of species abundance. We hypothesized that (H_1_) a long-term mesophication trend will be evident from temporal changes in relative abundances of dominant taxa, and that the magnitude of these changes will differ among strata, with the understory experiencing the largest dominance shifts and the overstory changing gradually. We hypothesized (H_2_) a mesophication-driven homogenization trend, whereby the three recognized community types at the site [54] would become less distinct over time. Finally, we hypothesized (H_3_) that vegetation dynamics in the site would have a clear spatial component where localized shifts in species composition are linked to topography. We were particularly interested in the idea of environmental filtering of mesophytic species in the driest sites in the watershed.

## Methods

### Study Site

Permission for this study was granted by the Eastern Kentucky University Division of Natural Resources. Big Everidge Hollow (BEH) is a 52 ha stand of old-growth forest within the Lilley Cornett Woods Appalachian Research Station located on the Cumberland Plateau in Letcher County, KY (37° 05" N, 83° 00" W, Roxana Quadrangle). The climate in this region is temperate humid continental with cool winters, warm summers, and no distinct dry season [58]. Mean annual temperature and precipitation from 1900 to 2000 were 12.7°C and 119 cm, respectively [59]. There is no history of commercial timber harvest at the site, and anthropogenic impacts are minimal [60]. The topography of the site is varied, with north-, east-, and south-facing slopes ranging from 320 to 600 m a.s.l. in elevation, and the terrain is fairly steep, averaging 50% of vertical and reaching 90% in some areas [54]. Tree-ring analysis of the site’s fire history indicated a mean fire return interval of 9.3 (±10.9 SD) years and a period of increased fire frequency ca. 1870–1950 [61]. Detectable disturbance events (growth releases) occurred every ca. 5 years over the last ca. 300 years [61], and deposition of coarse woody debris was similar across topographic positions and decades [62].

### Data Collection

Muller [54] established 80 circular permanent survey plots (0.04 ha each) throughout the watershed in 1979 using a random stratified sampling design. The watershed was divided into 8 topographic strata based on aspect (north-, east-, and south-facing) and elevation (upper, middle, and lower), and within each stratum 10 plots were randomly established. A lower, east-facing set of plots could not be established due to topography of the watershed. Muller [54] also classified plots into distinct overstory community types based on the dominant overstory species found there: chestnut oak (*n* = 32), beech (*n* = 31), and mixed mesophytic (*n* = 17).

These 80 plots were used in repeated sampling of the overstory community (woody stems ≥ 2.5 cm DBH) in 1979, 1989, 1999, and 2010. In 1999, one of lower, south-facing plots (beech community type) could not be relocated; therefore data do not exist for this plot in that year. Data for this plot were removed from the datasets for the other three sampling years in order to maintain consistent sample sizes (*n* = 79). Living tree species stems were assigned to one of three size classes based on DBH: understory (2.5–9.99 cm), midstory (10–24.99 cm), and overstory (> 25 cm). These size class designations are a combination of the size divisions used in previous site analyses done by McEwan and Muller [52] and Muller [54], and are similar to other studies [34,63].

### Analysis

Dominant taxa of interest were *A*. *saccharum*, *A*. *rubrum*, *Carya* spp. (pooled *C*. *glabra* (Miller) Sweet, *C*. *cordiformis* (Wagenh.) K. Koch, *C*. *ovata* (Miller) K. Koch, *C*. *tomentosa* (Poiret) Nutt.), *Fagus grandifolia* Ehrh., *Liriodendron tulipifera* L., *Quercus alba* L., *Q*. *montana* Willd., *Q*. *rubra* L., minor *Quercus* spp. (*Q*. *coccinea* Muenchh., *Q*. *muehlenbergii* Englem., and *Q*. *velutina* Lam. pooled together due to low occurrence), and *Tsuga canadensis* Carrière. For each sampling year, the sitewide and plot-level relative stem density and basal area were calculated for each dominant taxon and for all other species pooled together within each size class (understory, midstory, overstory, and all stems together). Paired t-tests were used to detect significant changes in plot-level relative abundances between 1979 and 2010. The frequency of each dominant taxon was calculated as the % of plots in which it occurred, and the locations of presence were mapped in order to assess spatial patterns of distribution over time for each size class.

Nonmetric multidimensional scaling (NMDS; 50 random starts, maximum 100 runs each to avoid possible local minima) was used to assess changes in community composition using community matrices weighted by presence-absence, stem density, and basal area. The input distance matrices for NMDS were calculated using Jaccard distance for presence-absence data and Bray-Curtis dissimilarity for the abundance data. All ordinations utilized 3 axes, which gave the greatest reduction in stress (final values ranged 0.10–0.15) beyond which additional axes did not offer improvement. NMDS was performed using *metaMDSiter()* in the ‘vegan’ package in R [64,65]. Ordinations were followed by ADONIS to test for significant separation of plots based on the three pre-designated community types using *adonis()* in the ‘vegan’ package.

After reducing the dimensionality of the data with NMDS, Procrustes analysis (9,999 permutations) was used to compare the community ordinations between years (1979–1989, 1979–1999, and 1979–2010) within each size class. Procrustes analysis superimposes one ordination on another, using dilation, scaling, and rotation to achieve the best fit by minimizing the sum of squared residuals (*m*^2^) [66]. ProTest with 999 permutations was used to test the significance of each Procrustean fit and obtain correlation coefficient values (*r*) [67]. ProTest is a permutation procedure that tests whether the concordance between the two ordinations is significantly different from that of randomly generated matrices. This was done using *procrustes()* and *protest()* in the ‘vegan’ package in R [64].

Each Procrustes fit provides average residual values for each plot, of which higher values indicate greater distance between related points (plots) in the ordinations (interpreted as the magnitude of compositional change). The interactive effect of elevation, aspect, and slope on relative moisture availability across the site was approximated using a modified version of Parker’s Topographic Relative Moisture Index [68]. Each topographic variable was rescaled so that values ranged from 0 to 2 (where south-facing, high elevation, and greater steepness represent drier conditions with values closer to 0), and an overall moisture value was calculated for each plot using the following equation:

Moisture=[(Elevation+Aspect+(Slope*0.5))/5]*100

Slope steepness was assigned half the weight of aspect and elevation due to its perceived lesser influence on local water availability. Linear regression was used to test for relationships between Procrustes residuals and the calculated moisture index values.

## Results

### Relative Abundance

**Fig 1:**
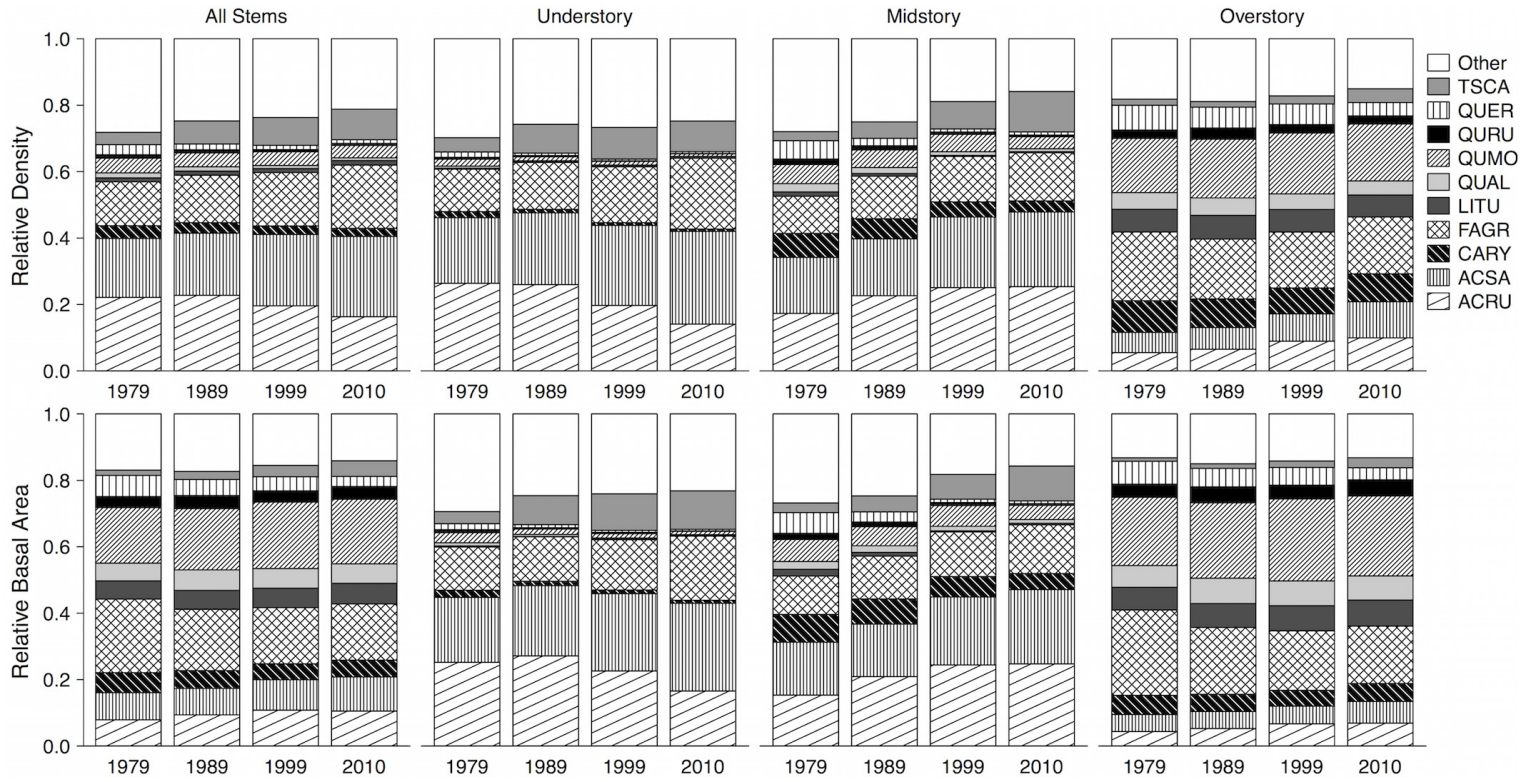
Relative abundances of dominant taxa over time. Relative density (stems m^-2^) and basal area (m^2^ ha^-1^) of dominant taxa in BEH: *Tsuga canadensis* (TSCA), *Quercus alba* (QUAL), *Q*. *montana* (QUMO), *Q*. *rubra* (QURU), minor *Quercus* species (*Q*. *coccinea*, *Q*. *muehlenbergii*, and *Q*. *velutina*; QUER), *Liriodendron tulipifera* (LITU), *Fagus grandifolia* (FAGR), *Carya* spp. (CARY), *Acer saccharum* (ACSA), and *A*. *rubrum* (ACRU).

Within each of the strata, the general patterns of relative density and basal area across the selected taxa were quite similar to each other; however, the patterns exhibited when all stems were considered together differed between the two abundance measures. The all-stem relative densities reflected the patterns of *Acer* dominance seen in the understory and midstory, whereas the all-stem relative basal area values reflected the *Quercus*-dominated pattern seen in the overstory (Fig 1). Considering all stems together, there were overall significant increases in the relative density of *A*. *saccharum* (*P* = 0.00022), *F*. *grandifolia* (*P* = 0.0049), and *T*. *canadensis* (*P* = 0.000063), and significant increases in the relative basal area of *A*. *saccharum* (*P* = 0.031), *A*. *rubrum* (*P* = 0.017), and *T*. *canadensis* (*P* = 0.0012; S1 Table). There were overall decreases in the relative density of *A*. *rubrum* (*P* = 0.024), *Carya* spp. (*P* = 0.011), *Q*. *alba* (*P* = 0.013), *Q*. *rubra* (*P* = 0.00023), and the combined minor *Quercus* species (*P* = 0.000081). The only decrease in all-stem relative basal area occurred in the minor *Quercus* spp. group (*P* = 0.0017; S1 Table).

Significant increases in relative abundance were generally associated with mesophytic species (S1 Table). *Acer saccharum* significantly increased in relative density in all three strata (understory *P* = 0.012; midstory *P* = 0.0000044; overstory *P* = 0.034), but relative basal area only increased in the understory (*P* = 0.039) and midstory (*P* = 0.0000013). *Acer rubrum* significantly increased in relative abundance in the midstory (density *P* = 0.00052; basal area *P* = 0.0089) and overstory (density *P* = 0.0012; basal area *P* = 0.033), but decreased in relative density in the understory (*P* = 0.00033). Relative abundance of understory *F*. *grandifolia* significantly increased over time (density *P* = 0.0004; basal area *P* = 0.014), and there was a slight decrease in relative basal area in the overstory (*P* = 0.046). The relative density of *T*. *canadensis* significantly increased in the understory (*P* = 0.0028) and midstory (*P* = 0.0012), and relative basal area increased in all three strata (understory *P* = 0.00026; midstory *P* = 0.0054; overstory *P* = 0.030)

Significant decreases in relative abundance were generally seen among *Carya* spp. and *Quercus* species, and most of these decreases occurred in the smaller stem classes (S1 Table). Significant decreases in relative density and basal area were seen for *Carya* spp. in the understory (*P* = 0.0051 and 0.045, respectively) and midstory (*P* = 0.045 and 0.017). All understory *Quercus* decreased in relative density and basal area over time (*Q*. *montana*, *P* = 0.0019 and 0.0043; *Q*. *rubra*, *P* = 0.010 and 0.0033; minor *Quercus* spp., *P* = 0.021 and 0.023), and we were alarmed to find that no understory *Q*. *alba* individuals were recorded in any of the sampling plots in 2010. In the midstory, *Q*. *rubra* and minor *Quercus* spp. significantly decreased in both relative density (*P* = 0.016 and 0.000054, respectively) and relative basal area (*P* = 0.032 and 0.00042), and *Q*. *alba* decreased in relative density only (*P* = 0.0078). In the overstory, only the combined minor *Quercus* spp. showed significant decreases in relative density (*P* = 0.029) and basal area (P = 0.021).

### Spatial Patterns of Frequency

**Fig 2:**
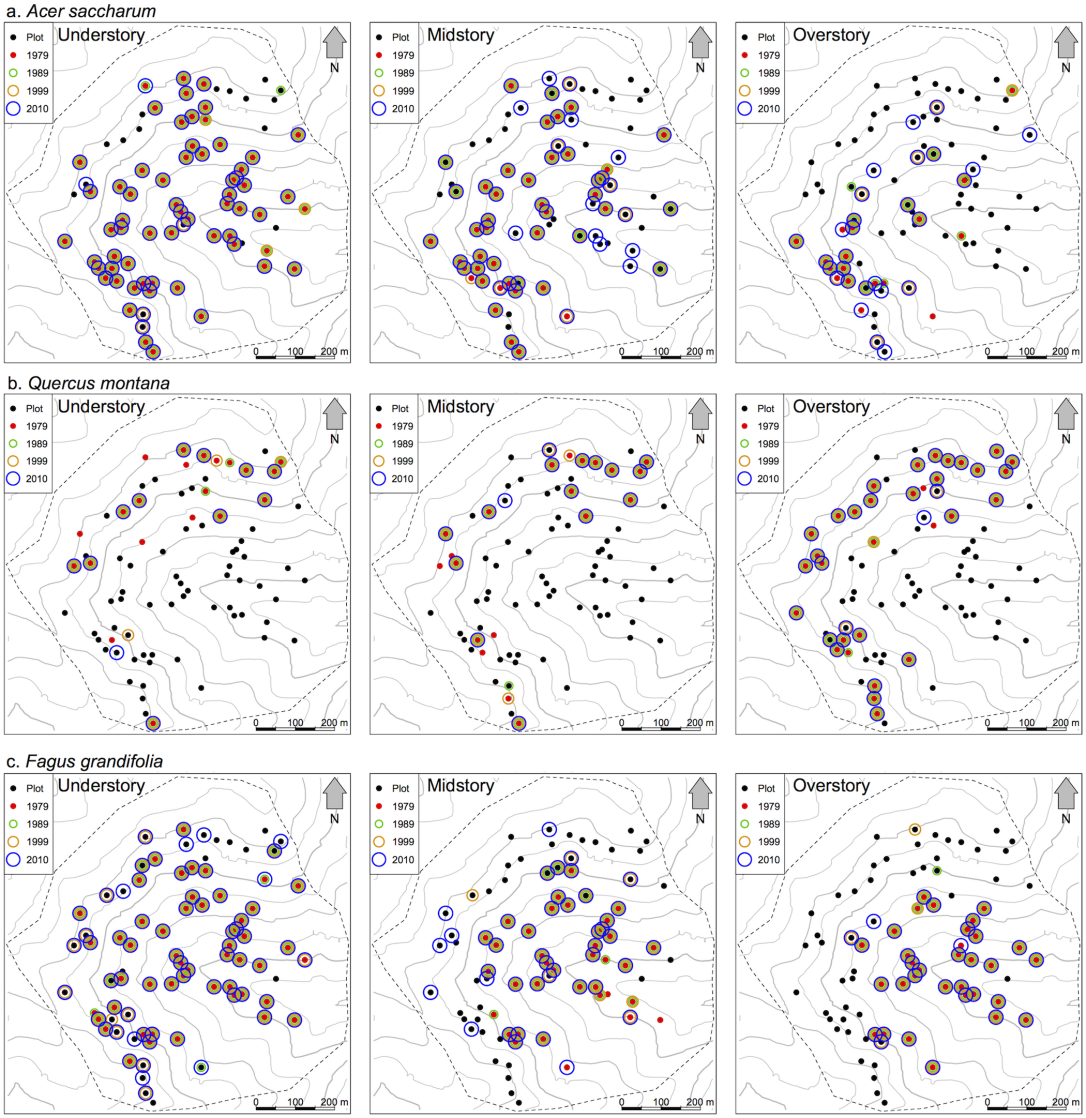
Spatial frequency of select dominant taxa. Presence of (a) *Acer saccharum*, (b) *Quercus montana*, and (c) *Fagus grandifolia* in plots across four sampling years. Colored symbols indicate presence of at least one individual in the plot for the corresponding year: 1979 (red), 1989 (green), 1999 (orange), 2010 (blue).

The spatial ranges of understory and midstory stems of *Quercus* and *Carya* appeared to be contracting, with continual regeneration and recruitment restricted to upper elevation areas, especially on the south-facing slope of the watershed (Fig 2b, S1 Fig). Overstory stems of both taxa were consistently present across all aspect positions (north-, south-, and east-facing slopes), but were generally absent from the lowest elevation plots (Fig 2b, S1 Fig). Understory *Q*. *montana* and *Carya* spp. were approximately half as frequent in 2010 compared to 1979, while losses of understory *Q*. *alba* and *Q*. *rubra* were more substantial (S2 Table). Midstory *Quercus* and *Carya* spp. all decreased in frequency, with *Q*. *rubra* and the minor *Quercus* species experiencing the greatest reductions. Overstory *Q*. *alba*, *Q*. *rubra*, and *Carya* spp. increased slightly in frequency over the thirty-year period, while *Q*. *montana* maintained its frequency and the minor *Quercus* species (*Q*. *coccinea*, *Q*. *muehlenbergii*, *Q*. *velutina* together) showing a distinct, but slight, decrease (S2 Table). *Liriodendron tulipifera* showed similar trends to those of *Quercus* and *Carya*, where understory and midstory individuals were less frequent than overstory trees and decreased in frequency over time (S2 Table).

The spatial distributions of the two *Acer* species strongly overlapped in the understory, and both were consistently present across >70% of plots over the 30-year period (Fig 2a, S2a Fig, S2 Table). Both species became more frequent in the midstory and overstory, but *A*. *saccharum* had a strong presence on the more mesic north-facing slope, while *A*. *rubrum* seemed to have greater establishment on the drier south-facing slope, especially at high elevations (Fig 2a, S2a Fig). The frequency of *F*. *grandifolia*, another mesophytic species, increased in the understory and midstory (S2 Table), expanding into upper elevation plots (Fig 2c). Overstory *F*. *grandifolia* stems were consistently restricted to low and mid elevation plots, with a frequency of up to 43% of plots (Fig 2c). *Tsuga canadensis* increased in frequency among all three strata (S2 Table) with understory and midstory individuals becoming more frequent on the east- and south-facing slopes, but still absent from the mid to high elevation areas of the north-facing slope. Overstory *T*. *canadensis* were restricted to the mesic cove habitats in lowest parts of the watershed (S2c Fig).

### Community-scale Dynamics (NMDS and Procrustes)

Regardless of how the ordinations were weighted (presence-absence, density, or basal area), Procrustean comparisons of 1979 and 2010 generally yielded the lowest *r* values, indicating the least amount of agreement between ordinations for these two years (Table 1). The one exception was the comparison of overstory stem communities based on basal area where 1979 and 1999 had the least agreement (*r* = 0.743). The correlation coefficients obtained from comparisons using all stems together were generally higher than those obtained from comparisons based on each of the three strata (Table 1). All ProTest outcomes were significant (*P* = 0.001), which was expected because the same set of plots were compared through time, and it was assumed they would have significant concordance.

**Table 1:** Results of ProTest significance tests of temporal Procrustes comparisons. Procrustean comparisons were made between years for all stems (≥2.5 cm dbh), understory (2.5–9.99 cm dbh), midstory (10–24.99 cm dbh), and overstory (≥25 cm dbh) using NMDS ordinations weighted by presence-absence, stem density, or basal area. Significance of comparisons was estimated using ProTest (999 permutations); correlation coefficients, *r*, are reported.

	Presence/Absence		Density		Basal Area	
Comparison	*r*	*P*	*r*	*P*	*r*	*P*
All Stems						
1979–1989	0.844	0.001	0.838	0.001	0.943	0.001
1979–1999	0.768	0.001	0.909	0.001	0.909	0.001
1979–2010	0.771	0.001	0.818	0.001	0.878	0.001
Understory						
1979–1989	0.812	0.001	0.778	0.001	0.768	0.001
1979–1999	0.760	0.001	0.854	0.001	0.742	0.001
1979–2010	0.675	0.001	0.664	0.001	0.666	0.001
Midstory						
1979–1989	0.739	0.001	0.715	0.001	0.749	0.001
1979–1999	0.720	0.001	0.707	0.001	0.687	0.001
1979–2010	0.577	0.001	0.653	0.001	0.640	0.001
Overstory						
1979–1989	0.815	0.001	0.757	0.001	0.828	0.001
1979–1999	0.774	0.001	0.728	0.001	0.743	0.001
1979–2010	0.757	0.001	0.696	0.001	0.798	0.001

**Fig 3:**
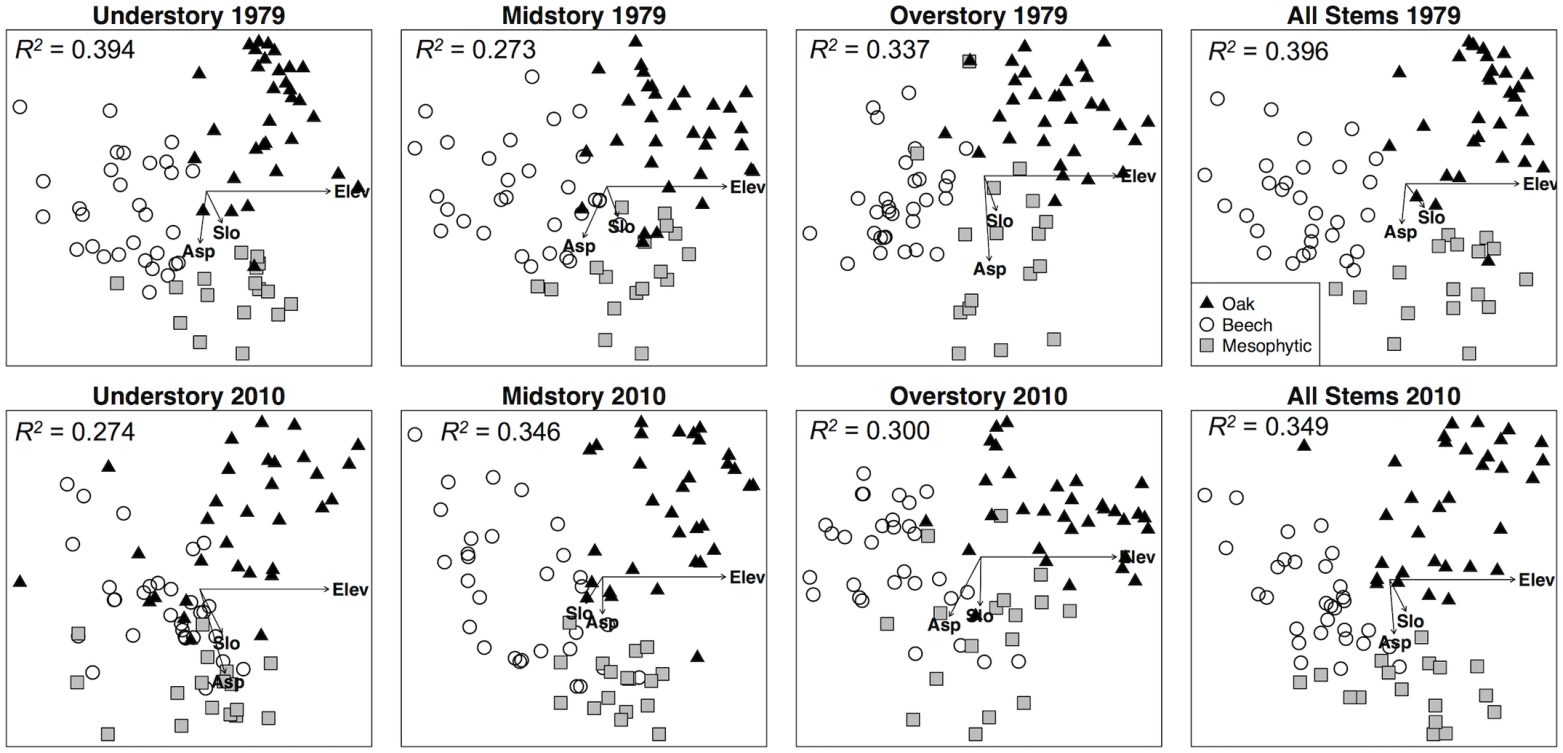
Community ordinations of forest strata for 1979 and 2010. NMDS ordinations of plots weighted by density (stems ha^-1^) for each stratum (understory, midstory, overstory) as well as all data together. Symbols represent overstory community types as designated by Muller (1982): Chestnut oak (solid black triangle), Mixed Mesophytic (grey square), and Beech (open circle).

There was significant distinction among the three community types designated by Muller (1982) within each dataset (ADONIS *P* = 0.001). In general, the amount of variation in the NMDS ordinations explained by the community types decreased over time (ADONIS *R*^2^ values decreased; S3 Table), suggesting that these communities are becoming less distinct. The exceptions were the midstory communities weighted by stem density and basal area, which had an overall increase in explained variance (ADONIS *R*^2^) over time (S3 Table). Comparison of NMDS plots for 1979 and 2010 revealed a pattern where the three community types were maintained most distinctively in ordinations based on all stems sampled (Fig 3, S3 and S4 Figs). An increased overlap between communities (indicating convergence of community types) was only apparent when inspecting ordinations based on any of the three strata (Fig 3, S3 and S4 Figs), and this trend was strongest in the understory.

There were some significant relationships between temporal shifts in plot composition (as measured by Procrustes residuals) and local moisture availability, all of which indicated that plots located in moister areas of the watershed experienced greater compositional shifts (*P* < 0.05; Table 2). These significant relationships were generally weak (*r*^2^ < 0.2) and were not consistently present across abundance weighting methods (presence-absence, stem density, basal area; except the understory for the 1979–1999 interval) or with increasing time intervals within the strata. This positive moisture-composition shift association was most frequent in the understory stratum, occurred occasionally in the midstory and on a sitewide scale (all stems), and was not found at all in the overstory (Table 2).

**Table 2:** Relationships between temporal community change and site moisture. Results of linear regression between Procrustes residuals and the topographic moisture index generated for the site. Bold values indicate a statistically significant relationship (*P* < 0.05).

	Presence/Absence		Stem Density		Basal Area	
	*r*^2^	*P*	*r*^2^	*P*	*r*^2^	*P*
All						
1979–1989	0.025	0.086	**0.044**	**0.035**	-0.004	0.407
1979–1999	0.022	0.100	**0.167**	**0.0001**	-0.006	0.482
1979–2010	0.012	0.169	-0.012	0.751	-0.012	0.796
Understory						
1979–1989	0.020	0.109	**0.061**	**0.016**	**0.075**	**0.008**
1979–1999	**0.091**	**0.004**	**0.128**	**0.0007**	**0.119**	**0.001**
1979–2010	**0.069**	**0.011**	-0.0010	0.340	-0.006	0.469
Midstory						
1979–1989	0.027	0.079	0.018	0.121	**0.051**	**0.026**
1979–1999	0.014	0.149	0.007	0.211	-0.006	0.463
1979–2010	**0.047**	**0.031**	**0.053**	**0.023**	0.029	0.072
Overstory						
1979–1989	-0.003	0.391	-0.009	0.580	-0.012	0.813
1979–1999	-0.004	0.413	-0.001	0.349	-0.007	0.512
1979–2010	0.013	0.157	0.0006	0.310	0.001	0.302

## Discussion

Forest dynamics are known to be influenced by disturbance processes and shifting environmental conditions, and datasets that (a) provide long-term perspective and (b) allow for assessment of these dynamics across topographic gradients are highly valuable. Our study satisfies both of these conditions and is located in an old-growth stand with minimal anthropogenic influence. As such, our work provides an important assessment of regional baseline dynamics in eastern North American forests currently undergoing “mesophication” [24] and contributes to the larger body of work toward understanding long-term forest dynamics (e.g. [28,32,41,69–76]). Working on the same plots as the present study, McEwan and Muller [52] provided evidence of long-term oak-maple dynamics and spatially explicit patterns similar to other studies [32,55,77,78] where oak subcanopy stems are increasingly restricted to the driest areas of the watershed. Use of data covering a greater temporal extent including an added decade, and division of stems 2.5–25 cm dbh into two substrata (midstory and understory), allowed us to further examine the temporal and spatial dynamics in this old-growth forest. We hypothesized (H_1_) that the relative abundances of dominant taxa would shift through time and this pattern would manifest differently among forest strata. As was reported by McEwan and Muller [52] and in myriad other studies [25–45] we found that mesophytic species (*Acer*, *Fagus*, *Tsuga*) became more abundant over 30 years while *Quercus* and *Carya* experienced the opposite, especially within the smaller size classes. The widespread abundance of juvenile *A*. *saccharum*, *A*. *rubrum*, and *F*. *grandifolia* in BEH confers a recruitment advantage to these species [21,23]. Simply having more young individuals available for recruitment increases a species’ chances of successfully capturing a canopy gap, and for mesophytic species, the prevailing humid climate and lack of fire regime [46] further bolsters this numbers advantage. Studies of logged stands have described an accelerated oak-to-maple succession that is thought to be predictive of such a long-term outcome for less disturbed areas [35,57,79,80]. Natural canopy gap formation is the main source of disturbance at our site, and the resultant ongoing, stochastic recruitment coupled with current climate conditions could facilitate a slow shift in site-wide overstory composition unfolding over decades or centuries in the absence of major disturbance [6–8,81].

The native tree species of eastern North America have coexisted in the landscape for thousands of years, persisting through fluctuations in climate conditions and disturbance regimes [82,83]. For any given species, favorable periods allow high survival and recruitment of individuals, and the resulting mature trees act as long-lived reservoirs of reproductive potential that carry the species through periods of unfavorable conditions. This storage effect [84] is evident in BEH as the present overstory dominance of shade-intolerant species points toward historic site conditions more favorable to their regeneration and recruitment than for mesophytic species. In particular, the abundance of overstory *L*. *tulipifera*, coupled with its current lack of regeneration, points toward a historical period of greater disturbance relative to recent decades [27,33,43]. Large overstory individuals of *Quercus*, *Carya*, and *Liriodendron* have continued to coexist with mesophytic species, even though they are currently recruitment-limited [19] in some areas of BEH, as evidenced by a lack of younger individuals in some plots containing overstory trees assumed capable of producing viable seed based on previous seedling inventories [51,53]. We hypothesized (H_2_) that there would be compositional homogenization of the three community types through time as mesophytic species come to dominate the watershed (i.e., “mesophication” [24,85,86]). This pattern was seen clearly in the understory but appeared to be driven by the decreasing presence of *Quercus* and *Carya* in the lower strata, as opposed to increased spatial dominance of mesophytic species, which were already highly abundant and widespread in 1979. Aldrich et al. [87] showed a similar result where mesophytic species greatly expanded in spatial distribution from 1926 to 1976 through high recruitment of small stems but *Quercus* persisted through time as large trees, and McDonald et al.’s [88] study exhibited similar trends from 1949 to 1997. If environmental conditions continue to favor mesophytic species recruitment, we expect that mesophication of community types will propagate through the midstory and overstory strata as well.

The influence of topographic position on local species distributions via microclimatic variation has long been recognized as a driver of spatial patterns of vegetation in forests [89–92]. We found moderate support for our third hypothesis (H_3_) that species composition and dynamics would be linked to topography, especially in the driest portions of the watershed where we expected mesophytic species to have limited regeneration success. In our study forest there are distinct spatial patterns of overstory *A*. *saccharum*, *Q*. *montana*, *F*. *grandifolia*, and *T*. *canadensis* that align with the topography in a way that suggests habitat filtering is an historically important driver of these species’ realized niches at the site. As an example, *T*. *canadensis* overstory stems are found in the lowest elevation portions of the watershed along the streams while understory stems have a much broader spatial distribution (Supporting Information). We found that *F*. *grandifolia* and *A*. *saccharum* are able to establish in the understory in some of the most xeric areas of the watershed; however this environment may not be suitable for their long-term survival [17,93]. Although *Q*. *alba* overstory distribution is relatively widespread in the watershed, regeneration was spatially limited to high elevation south-facing portions of the watershed. These dry areas of the watershed are, ostensibly, serving as refugia for *Quercus*, and some *Carya*, as they are becoming restricted to local environments with the most suitable conditions [94–97], least competition from mesophytic species, or both. The clear and persistent separation of a large number of chestnut oak community plots in our NMDS ordinations is consistent with this phenomenon, and other studies have shown a trend of continued *Quercus* dominance on ridgetops and south-facing slopes [32,55,77,78,88].

We posit that there are certain areas of the watershed where edaphic factors create habitat suitable for a particular overstory community type. We recognize these areas as stable site habitats: upper south-facing slope, upper north-facing slope, and lower mesic cove. We hypothesize that transitional, or labile, habitat zones exist between these areas where the local environmental is more susceptible to the influence of prevailing climate and disturbance regimes. For example, it is clear that the upper south-facing slope is the most suitable habitat for *Quercus* species at our site, but large individuals are also found across other aspect positions and at lower elevations. Regeneration of *Quercus* in these “labile” habitats is failing–a regionally cool, wet climate, lack of fire disturbance, or other environment factors have shifted in a way that conditions are no longer locally favorable to oaks. At the same time, these conditions have allowed abundant and widespread regeneration of shade-tolerant, mesophytic species. Many forests are now undergoing the successional changes similar to the findings of our study [e.g. 28,33,41,45,72,74], and understanding long-term patterns is important for prediction of future influences on wildlife populations, future disturbance regimes, and both carbon and nutrient cycling [98–105]. For example, increased abundance of *Acer* species may alter nitrogen cycling dynamics [98,101] and create cooler, moister local microclimates that can influence the composition of understory vegetation [104]. Loss of *Quercus* species could substantially influence the population dynamics of wildlife species including numerous game birds and mammals that rely on acorns as a food source [102]. The wide-ranging implications associated with long-term meosphication dynamics emphasizes the need for continued, long-term monitoring of eastern North American forests.

The spatial ebb and flow of species indicated by the patterns in our long-term data set suggest tension between fluidity of lottery-model understory regeneration based on short-term local conditions and stability created by storage effects as long-lived canopy trees maintain dominance through changes in environmental conditions wherein their seedlings may be non-competitive over the short-term. The watershed-scale outcome thus far, then, has been a suite of species able to coexist despite long-term fluctuations in climate and disturbance regime. Documentation of baseline dynamics will become even more valuable in the face of anthropogenic climate change and the impending introduction of pests and pathogens, including hemlock woolly adelgid, emerald ash borer, and beech bark disease, that have the potential to decimate adult tree populations of particular species. As climate-based environmental conditions shift and large-scale species die-offs substantially open the forest canopy, the interplay between these complex ecological relationships will almost certainly be substantially altered.

## Supporting Information

S1 Table Sitewide relative abundance (density, stems ha^-1^; basal area, m^2^ ha^-1^) of dominant taxa over 30 years in Big Everidge Hollow. Values are percentages. Asterisks indicate significant plot-level differences between 1979 and 2010 (paired t-test).
(PDF)

S2 Table Plot frequencies (%) of dominant taxa over 30 years in Big Everidge Hollow, an old-growth stand in southeastern Kentucky (*n* = 79). *Carya* spp. includes *C*. *cordiformis*, *C*. *glabra*, *C*. *ovata*, and *C*. *tomentosa*. Minor *Quercus* spp. include *Q*. *coccinea*, *Q*. *muehlenbergii*, and *Q*. *velutina*.
(PDF)

S3 Table Results of ADONIS testing for significant separation among overstory community type designations (Muller 1982) in BEH. Analyses based on community data weighted by presence-absence, stem density, and basal area.
(PDF)

S1 Fig Spatial frequency of select dominant taxa. Presence of (a) *Quercus alba*, (b) *Quercus rubra*, and (c) *Carya* spp. in plots across four sampling years. *Carya* spp. includes *C*. *glabra*, *C*. *tomentosa*, *C*. *ovata*, *C*. *cordiformis*. Colored symbols indicate presence of at least one individual in the corresponding year: 1979 (red), 1989 (green), 1999 (orange), and 2010 (blue).
(TIFF)

S2 Fig Spatial frequency of select dominant taxa. Presence of (a) *Acer rubrum*, (b) *Liriodendron tulipifera*, and (c) *Tsuga canadensis* in plots across four sampling years. Colored symbols indicate presence of at least one individual in the corresponding year: 1979 (red), 1989 (green), 1999 (orange), and 2010 (blue).
(TIFF)

S3 Fig Community ordinations of forest strata for 1979 and 2010. NMDS ordinations of plots weighted by basal area (m^2^ ha^-1^) for each stratum (understory, midstory, overstory) as well as all data together. Symbols represent overstory community types as designated by Muller (1982): Chestnut oak (solid black triangle), Mixed Mesophytic (grey square), and Beech (open circle).
(TIFF)

S4 Fig Community ordinations of forest strata for 1979 and 2010. NMDS ordinations of plots weighted by presence-absence for each stratum (understory, midstory, overstory) as well as all data together. Symbols represent overstory community types as designated by Muller (1982): Chestnut oak (solid black triangle), Mixed Mesophytic (grey square), and Beech (open circle).
(TIFF)

S1 Dataset Overstory and topographic data. (XLSX)
