# Supplementary material for: Thirty Years of Compositional Change in an Old-Growth Temperate Forest: The Role of Topographic Gradients in Oak-Maple Dynamics
Source: PLoS One. 2016 Jul 28;11(7):e0160238. doi: 10.1371/journal.pone.0160238 (PMC4965133; doi:10.1371/journal.pone.0160238)
Supplement: S1 Table — Values are percentages. Asterisks indicate significant plot-level differences between 1979 and 2010 (paired t-test). (PDF) [file pone.0160238.s001.pdf]

S1 Table.

	All Stems ≥2.5 cm dbh				Understory Stems 2.5–10 cm dbh				Midstory Stems 10–25 cm dbh				Overstory Stems >25 cm dbh			
**Relative Density**	**1979**	**1989**	**1999**	**2010**	**1979**	**1989**	**1999**	**2010**	**1979**	**1989**	**1999**	**2010**	**1979**	**1989**	**1999**	**2010**
Acer rubrum	22.16	22.77	19.56	16.30*	26.32	25.97	19.67	14.03**	17.30	22.66	25.03	25.37**	5.45	6.51	8.88	9.97*
Acer saccharum	17.70	18.68	21.50	24.18**	19.77	21.61	24.16	27.98*	16.87	17.14	21.25	22.43**	6.14	6.51	8.32	10.84*
Carya spp.	3.84	3.12	2.53	2.43*	1.87	1.02	0.77	0.69*	7.18	5.98	4.60	3.33**	9.55	8.61	7.75	8.39
Fagus grandifolia	13.25	14.36	16.10	19.05*	12.76	14.19	16.83	21.37**	11.21	12.85	13.69	14.50	20.68	18.07	16.82	17.13
Liriodendron tulipifera	1.19	1.18	1.21	1.35	0.28	0.16	0.44	0.52	1.31	0.79	0.31	0.39	6.82	7.14	6.81	6.64
Quercus alba	1.48	1.28	0.92	0.81*	0.60	0.33	0.11	0.00	2.50	1.80	1.12	0.88*	5.00	5.25	4.73	4.20
Quercus montana	4.41	4.22	4.17	3.78	2.11	1.22	1.03	0.77*	5.77	5.30	5.21	3.62	16.36	17.65	18.34	17.13
Quercus rubra	1.02	0.92	0.62	0.54**	0.60	0.33	0.22	0.12*	1.63	1.24	0.72	0.49*	2.50	3.36	2.46	2.45
Minor *Quercus* spp.	3.07	1.78	1.33	1.10**	1.59	0.73	0.52	0.52*	5.55	2.25	0.92	0.88**	7.50	6.30	6.24	4.02*
Tsuga canadensis	3.72	6.94	8.36	9.26**	4.33	8.68	9.54	9.19*	2.72	4.96	8.27	12.24*	1.82	1.68	2.46	4.20
Other	28.16	24.74	23.70	21.19	29.77	25.76	26.70	24.80	27.97	25.03	18.90	15.87	18.18	18.91	17.20	15.03
**Relative Basal Area**	**1979**	**1989**	**1999**	**2010**	**1979**	**1989**	**1999**	**2010**	**1979**	**1989**	**1999**	**2010**	**1979**	**1989**	**1999**	**2010**
Acer rubrum	7.90	9.36	10.82	10.53*	25.21	27.18	22.61	16.57	15.36	20.92	24.44	24.69*	4.37	5.28	6.67	6.93*
Acer saccharum	8.18	7.96	9.12	10.26*	19.59	21.14	23.31	26.44*	15.89	15.79	20.41	22.46**	5.15	5.11	5.35	6.48
Carya spp.	5.98	5.40	4.80	5.09	2.12	1.31	1.05	0.91*	8.38	7.53	6.20	4.78*	5.73	5.21	4.75	5.42
Fagus grandifolia	22.16	18.43	16.96	16.93	12.99	13.35	15.11	19.31*	11.55	12.95	13.35	14.68	25.73	20.11	17.96	17.30*
Liriodendron tulipifera	5.48	5.71	5.84	6.18	0.35	0.19	0.32	0.38	2.03	1.15	0.39	0.48	6.85	7.20	7.57	7.83
Quercus alba	5.34	6.21	5.92	5.83	0.96	0.52	0.23	0.00	2.33	2.00	1.39	1.14	6.52	7.63	7.44	7.26
Quercus montana	16.70	18.42	19.99	19.47	2.98	1.63	1.35	1.02*	6.66	5.66	6.24	4.29	20.56	22.70	24.69	24.07
Quercus rubra	3.34	3.92	3.35	3.89	0.96	0.40	0.36	0.12*	1.83	1.43	0.88	0.51*	3.95	4.77	4.17	4.89
Minor *Quercus* spp.	6.42	4.83	4.25	2.97*	1.81	0.95	0.60	0.49*	6.27	3.05	1.00	0.83**	6.89	5.54	5.31	3.61*
Tsuga canadensis	1.62	2.46	3.47	4.77*	3.62	8.73	10.99	11.64**	2.93	4.84	7.51	10.46*	1.10	1.42	1.94	3.05*
Other	16.87	17.31	15.47	14.07	29.41	24.59	24.08	23.12	26.77	24.67	18.19	15.70	13.16	15.02	14.17	13.15

Carya spp.: *C. cordiformis, C. glabra, C. ovata, C. tomentosa*. Minor *Quercus* spp.: *Q. coccinea, Q. muehlenbergii, Q. velutina*.
Other: *Acer pensylvanicum, Aesculus flava, Amelanchier arborea, Asimina triloba, Betula lenta, Carpinus caroliniana, Castanea dentata, Cercis canadensis, Cornus florida, Fraxinus americana, Hamamelis virginiana, Juglans nigra, Juniperus virginiana, Magnolia acuminata, M. macrophylla, Morus rubra, Nyssa sylvatica, Ostrya virginiana, Oxydendrum arboreum, Paulownia tomentosa, Pinus echinata, P. rigida, P. virginiana, Prunus serotina, Robinia pseudoacacia, Sassafras albidum, Tilia americana, Ulmus americana, U. rubra*
*$P < 0.05$; **$P < 0.001$
