# Supplementary material for: Thirty Years of Compositional Change in an Old-Growth Temperate Forest: The Role of Topographic Gradients in Oak-Maple Dynamics
Source: PLoS One. 2016 Jul 28;11(7):e0160238. doi: 10.1371/journal.pone.0160238 (PMC4965133; doi:10.1371/journal.pone.0160238)
Supplement: S2 Table — Carya spp. includes C. cordiformis, C. glabra, C. ovata, and C. tomentosa. Minor Quercus spp. include Q. coccinea, Q. muehlenbergii, and Q. velutina. (PDF) [file pone.0160238.s002.pdf]

S2 Table.

	All Stems ≥2.5 cm dbh				Understory Stems 2.5–9.99 cm dbh				Midstory Stems 10–24.99 cm dbh				Overstory Stems >25 cm dbh			
	**1979**	**1989**	**1999**	**2010**	**1979**	**1989**	**1999**	**2010**	**1979**	**1989**	**1999**	**2010**	**1979**	**1989**	**1999**	**2010**
Acer rubrum	88.6	87.3	87.3	87.3	79.7	73.4	74.7	73.4	63.3	69.6	73.4	74.7	22.8	26.6	35.4	43.0
Acer saccharum	79.7	79.7	83.5	83.5	78.5	78.5	81	79.7	46.8	53.2	62.0	72.2	21.5	22.8	25.3	35.4
Carya spp.	62.0	51.9	55.7	53.2	31.6	16.5	16.5	15.2	48.1	39.2	36.7	29.1	31.6	32.9	34.2	38.0
Fagus grandifolia	69.6	73.4	84.8	91.1	65.8	69.6	79.7	88.6	46.8	45.6	49.4	55.7	41.8	40.5	43.0	43.0
Liriodendron tulipifera	32.9	31.6	36.7	32.9	10.1	5.1	11.4	6.3	11.4	8.9	3.8	5.1	22.8	25.3	27.8	26.6
Quercus alba	39.2	36.7	35.4	31.6	11.4	5.1	2.5	0.0	22.8	16.5	12.7	11.4	21.5	24.1	26.6	22.8
Quercus montana	46.8	43.0	43.0	44.3	26.6	17.7	17.7	15.2	25.3	19.0	21.5	20.3	40.5	39.2	40.5	40.5
Quercus rubra	34.2	32.9	25.3	20.3	13.9	7.6	7.6	3.8	16.5	11.4	7.6	3.8	13.9	19.0	15.2	16.5
Minor *Quercus* spp.	43.0	30.4	26.6	24.1	13.9	6.3	3.8	6.3	32.9	15.2	8.9	5.1	25.3	22.8	22.8	19.0
Tsuga canadensis	22.8	31.6	36.7	40.5	21.5	27.8	35.4	30.4	8.9	12.7	20.3	31.6	6.3	8.9	8.9	11.4
