# Supplementary material for: Thirty Years of Compositional Change in an Old-Growth Temperate Forest: The Role of Topographic Gradients in Oak-Maple Dynamics
Source: PLoS One. 2016 Jul 28;11(7):e0160238. doi: 10.1371/journal.pone.0160238 (PMC4965133; doi:10.1371/journal.pone.0160238)
Supplement: S3 Table — Analyses based on community data weighted by presence-absence, stem density, and basal area. (PDF) [file pone.0160238.s003.pdf]

S3 Table.

	Presence/Absence		Density		Basal Area	
	R^2	P	R^2	P	R^2	P
Understory						
1979	0.350	0.001	0.394	0.001	0.376	0.001
1989	0.338	0.001	0.366	0.001	0.381	0.001
1999	0.298	0.001	0.316	0.001	0.331	0.001
2010	0.286	0.001	0.274	0.001	0.278	0.001
Midstory						
1979	0.308	0.001	0.273	0.001	0.249	0.001
1989	0.250	0.001	0.244	0.001	0.219	0.001
1999	0.284	0.001	0.334	0.001	0.296	0.001
2010	0.263	0.001	0.346	0.001	0.314	0.001
Overstory						
1979	0.378	0.001	0.337	0.001	0.332	0.001
1989	0.340	0.001	0.337	0.001	0.317	0.001
1999	0.312	0.001	0.301	0.001	0.300	0.001
2010	0.322	0.001	0.300	0.001	0.283	0.001
All Stems						
1979	0.352	0.001	0.439	0.001	0.396	0.001
1989	0.336	0.001	0.422	0.001	0.378	0.001
1999	0.325	0.001	0.410	0.001	0.376	0.001
2010	0.294	0.001	0.397	0.001	0.349	0.001
