# Supplementary figures and images for: Thirty Years of Compositional Change in an Old-Growth Temperate Forest: The Role of Topographic Gradients in Oak-Maple Dynamics
Source: PLoS One. 2016 Jul 28;11(7):e0160238. doi: 10.1371/journal.pone.0160238 (PMC4965133; doi:10.1371/journal.pone.0160238)

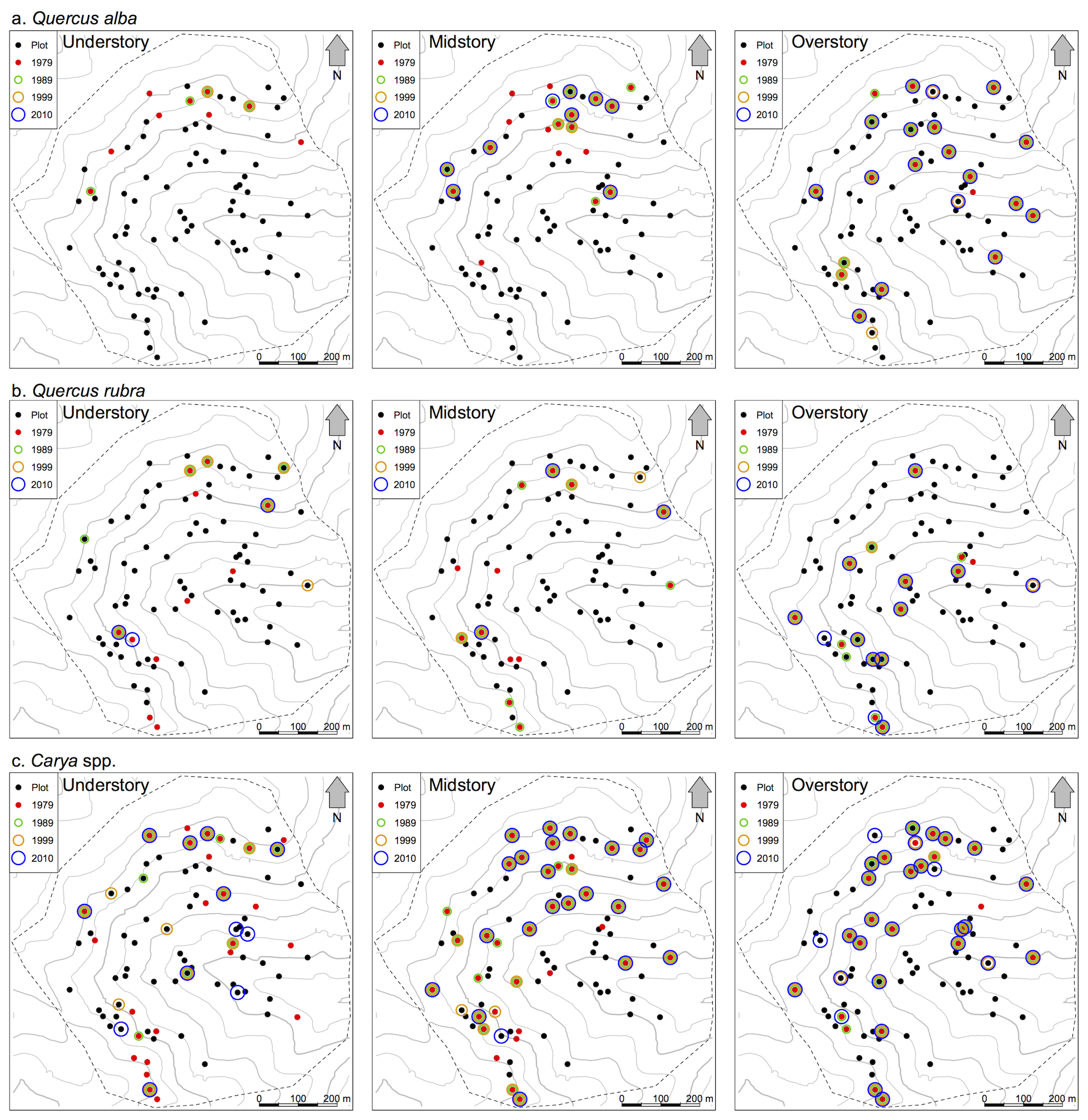

Supplement: S1 Fig — Presence of (a) Quercus alba, (b) Quercus rubra, and (c) Carya spp. in plots across four sampling years. Carya spp. includes C. glabra, C. tomentosa, C. ovata, C. cordiformis. Colored symbols indicate presence of at least one individual in the corresponding year: 1979 (red), 1989 (green), 1999 (orange), and 2010 (blue). (TIFF) [file pone.0160238.s004.tiff]

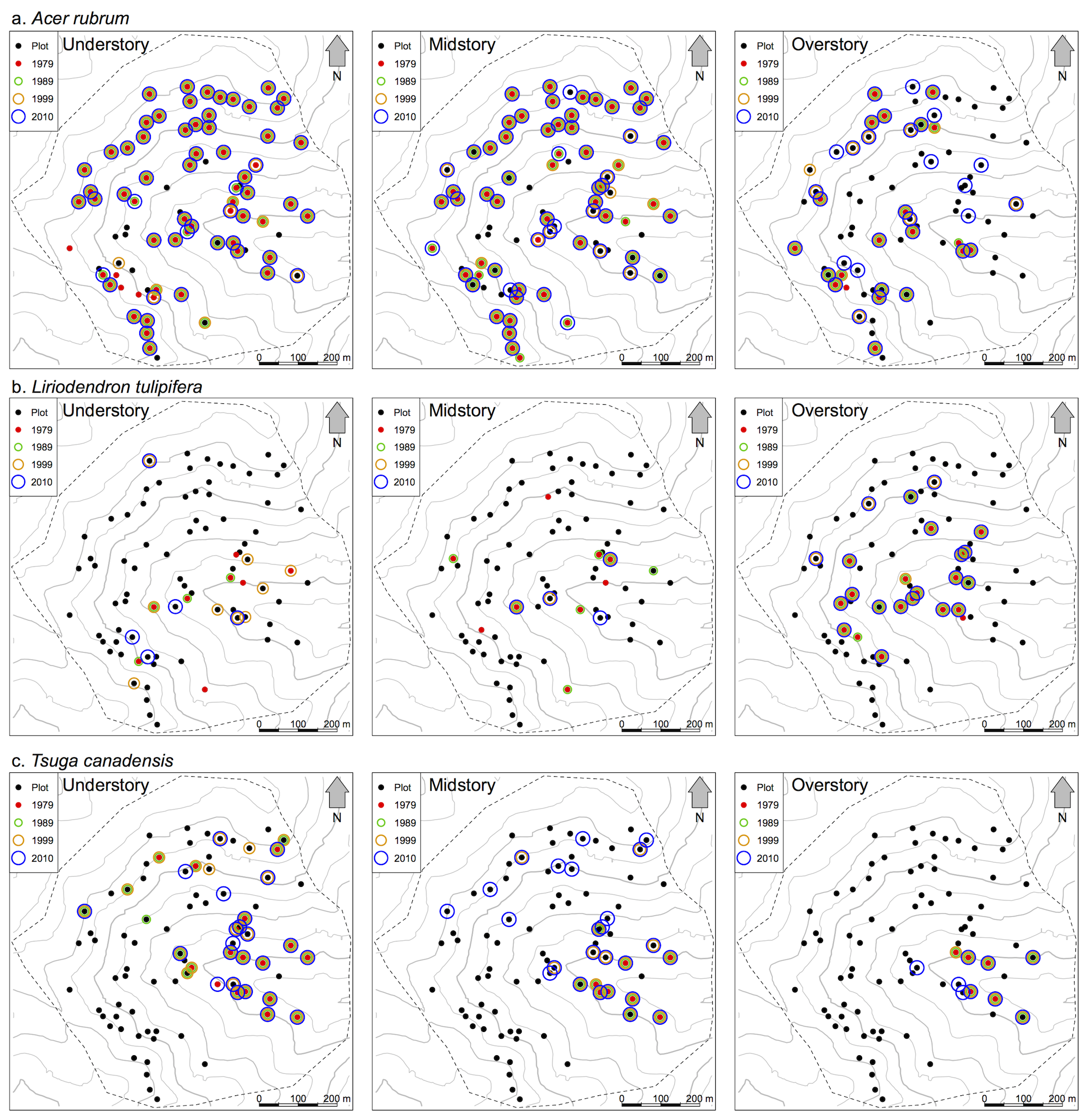

Supplement: S2 Fig — Presence of (a) Acer rubrum, (b) Liriodendron tulipifera, and (c) Tsuga canadensis in plots across four sampling years. Colored symbols indicate presence of at least one individual in the corresponding year: 1979 (red), 1989 (green), 1999 (orange), and 2010 (blue). (TIFF) [file pone.0160238.s005.tiff]

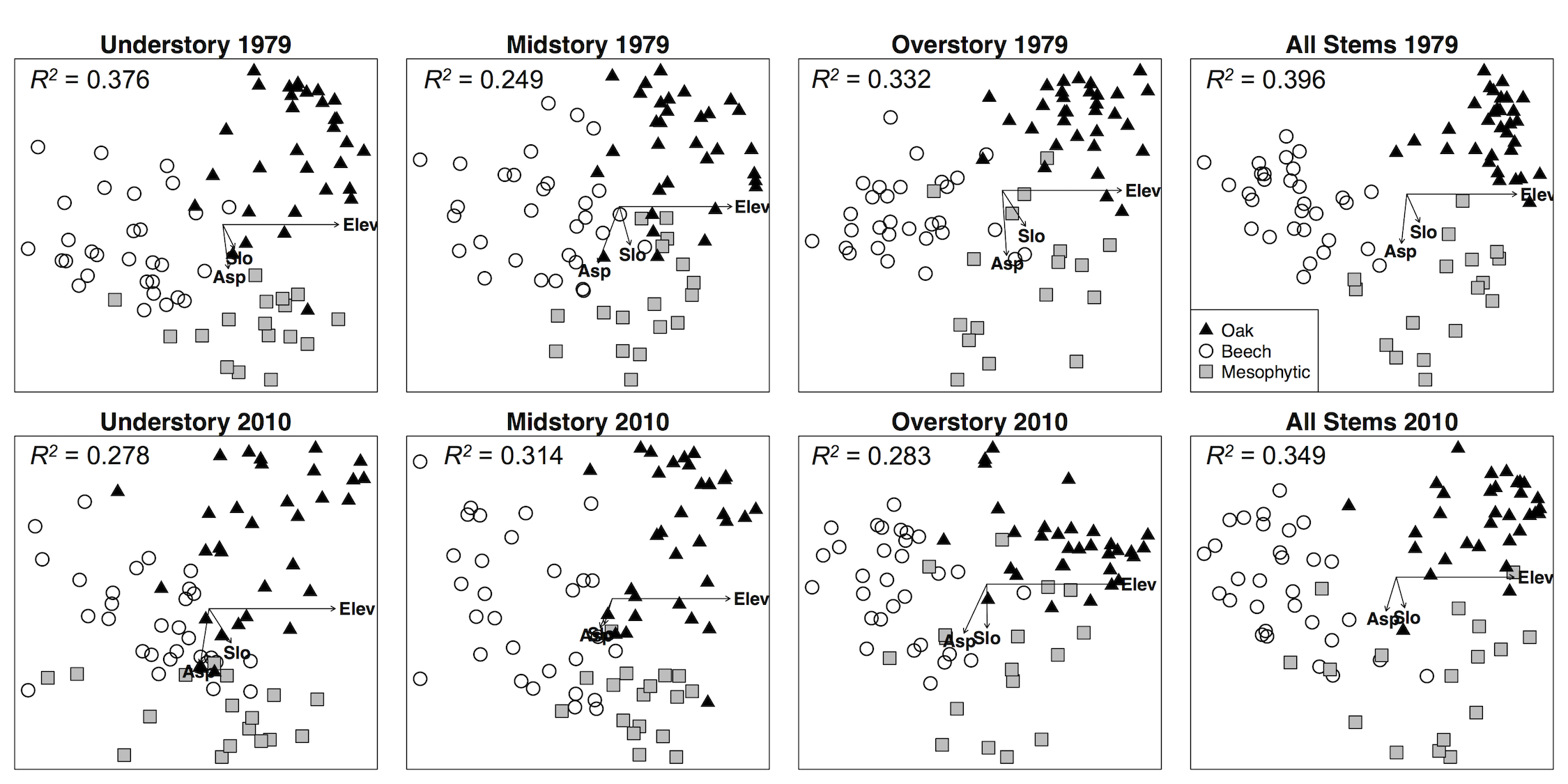

Supplement: S3 Fig — NMDS ordinations of plots weighted by basal area (m2 ha-1) for each stratum (understory, midstory, overstory) as well as all data together. Symbols represent overstory community types as designated by Muller (1982): Chestnut oak (solid black triangle), Mixed Mesophytic (grey square), and Beech (open circle). (TIFF) [file pone.0160238.s006.tiff]

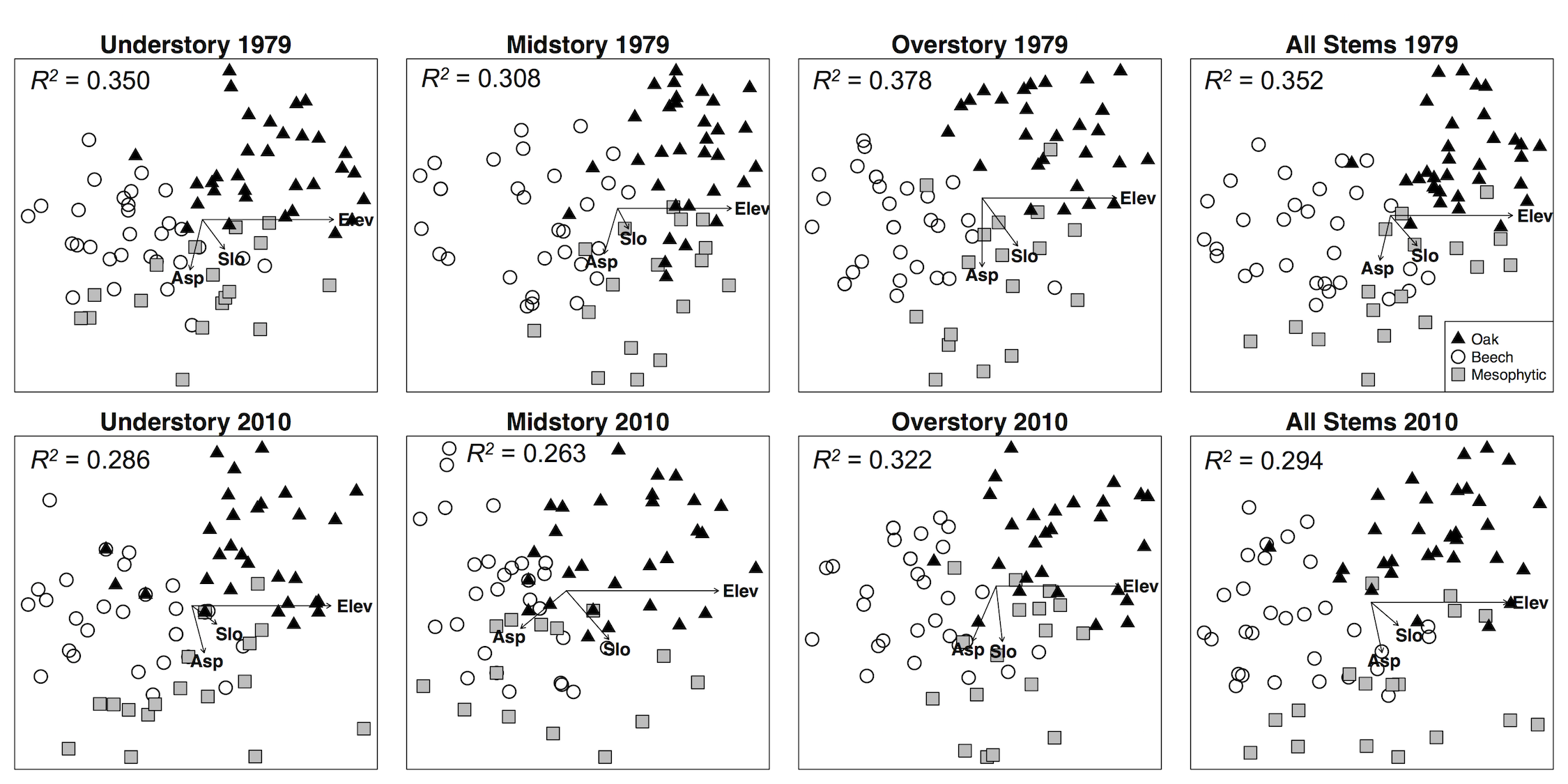

Supplement: S4 Fig — NMDS ordinations of plots weighted by presence-absence for each stratum (understory, midstory, overstory) as well as all data together. Symbols represent overstory community types as designated by Muller (1982): Chestnut oak (solid black triangle), Mixed Mesophytic (grey square), and Beech (open circle). (TIFF) [file pone.0160238.s007.tiff]
